# Finding More in Less: Precision Medicine for Pancreatic Cancer Using Residual Cytology Samples

**DOI:** 10.1111/cyt.70055

**Published:** 2026-01-26

**Authors:** Raquel Antón‐Peñalver, María Ortega, Jose Francisco González‐Muñoz, Desamparados Compañ, Constanza Galindo, Clara Arnau, Marisol Huerta, Valentina Gambardella, Desamparados Roda, Rosana Villagrasa, Vicente Sanchiz, Andrés Peña, Elena Muñoz‐Forner, Isabel Mora Oliver, Luis Sabater, Andrés Cervantes, Antonio Ferrández, Clara Alfaro‐Cervelló

**Affiliations:** ^1^ Departament de Patologia, Facultat de Medicina i Odontologia Universitat de València València Spain; ^2^ Servicio de Anatomía Patológica Hospital Clínico Universitario de Valencia, Instituto de Investigación Biomédica INCLIVA Valencia Spain; ^3^ Servicio de Oncología Médica, Hospital Clínico Universitario de Valencia, Instituto de Investigación Biomédica INCLIVA Universitat de València Valencia Spain; ^4^ Servicio de Medicina Digestiva Hospital Clínico Universitario de Valencia, Instituto de Investigación Biomédica INCLIVA Valencia Spain; ^5^ Unidad de Cirugía Hepato‐Biliopancreática, Servicio de Cirugía General y del Aparato Digestivo, Hospital Clínico Universitario de Valencia, Departamento de Cirugía Universitat de València, Instituto de Investigación Biomédica INCLIVA Valencia Spain; ^6^ Departamento de Medicina Universitat de València Valencia Spain

**Keywords:** cell block, liquid‐based cytology, microsatellite instability, next‐generation sequencing, pancreatic adenocarcinoma

## Abstract

**Background:**

Pancreatic ductal adenocarcinoma (PDAC) is one of the most lethal tumours and its incidence is increasing. Definitive diagnosis requires pathological confirmation. Cytology samples are obtained using endoscopic ultrasound‐guided fine needle aspiration (EUS‐FNA). The utility and storage conditions of residual liquid‐based cytology (LBC) for PDAC remain poorly defined.

**Methods:**

From 2020 to 2024, 268 patients were diagnosed with PDAC using EUS‐FNA and LBC. Cell blocks were prepared when possible, and residual LBC samples were stored.

**Results:**

Molecular studies were requested for 54 patients. Residual LBC was the only sample available in eight patients. Molecular analyses were requested at more than 2 months and up to 27 months after diagnosis in 31.7% of patients and performed on residual LBC or cell block based on availability and quality. Samples were not analysed in parallel. DNA concentration was higher in LBC than cell blocks, and MAPD (median absolute pairwise difference) was lower. LBC storage time did not affect NGS validity. Residual LBC provided an adequate proportion of valid studies in NGS DNA and MSI RT‐PCR, showing no difference to cell blocks. RNA provided fewer valid studies, with no differences between sample types. The most frequently detected mutations were *KRAS* (35/41) and *TP53* (12/41). No fusions or MSI were detected.

**Conclusions:**

In a real clinical setting, we show that residual LBC samples from PDAC are valid for molecular analysis, preserving the quality of nucleic acids. Residual LBC may be the only available sample and should be preserved to ensure patient access to targeted therapies.

## Graphical abstract

The study evaluates the use of residual liquid‐based cytology (LBC) samples from pancreatic ductal adenocarcinoma (PDAC) for molecular analysis. It shows that LBC samples preserve nucleic acid quality and remain suitable for next‐generation sequencing (NGS) and MSI testing even after prolonged storage, underscoring their value when other samples are unavailable for targeted therapy decisions.

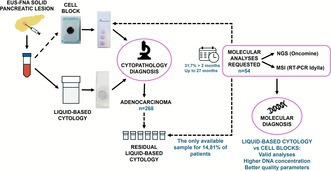

## 1 Introduction

Pancreatic ductal adenocarcinoma (PDAC) is one of the deadliest tumours, with a rising incidence [1]. Most cases are diagnosed at advanced stages, making them unresectable due to vascular invasion or distant metastasis. Molecular analysis has uncovered a range of potential targetable alterations in PDAC [2, 3], for which treatments with KRAS inhibitors, such as sotorasib or adagrasib for KRAS‐G12C, or MTRX1133 for p.G12D, present a promising strategy [4, 5, 6]. Although microsatellite instability (MSI) is rare in PDAC, treatment with immune‐checkpoint inhibitors might be an alternative for some patients [7].

The definitive diagnosis of PDAC requires pathological confirmation, which can sometimes be challenging. The most commonly used and recommended technique to obtain cytologic samples is endoscopic ultrasound‐guided fine needle aspiration (EUS‐FNA) [8]. Liquid‐based cytology (LBC) processing is widely employed and can also be used to obtain paraffin cell blocks. The yield of PDAC tumour cells is often low from both LBC and cell blocks, requiring methodological optimization to maximise pathological and molecular diagnosis. LBC samples preserve quality and permit direct DNA extraction [9]. Several studies have shown that residual LBC is a valid sample for molecular studies of lung cancer [10, 11, 12]. Another widely studied field is the use of residual LBC in cervical cytology, in which long‐term storage allows DNA extraction and human papillomavirus detection [13, 14]. However, molecular studies on cytological samples of PDAC have been sparsely reported.

Storage of residual LBC samples is currently recommended until pathological diagnosis is completed, that is, following additional molecular analyses, or later in case of malignancy [15]. Samples are preferably stored at 4°C and nucleic acids should be extracted promptly. Paraffin‐embedded cell block samples are usually stored longer, for at least 10 years [15].

In PDAC patients without available cell block or in whom surgery was not performed, residual LBC is the only available sample after diagnosis. As in clinical practice molecular diagnosis was sometimes requested by the oncologist months after diagnosis, we selected residual LBC samples diagnosed as PDAC and stored them longer to facilitate patient access to potential targeted therapies. Here, we retrospectively analyse the quality and results of molecular analyses (NGS and RT‐PCR for MSI) performed on residual LBC and cell block samples in our center.

## 2 Methods

### 2.1 Patients

This study reviewed results from cytologic and molecular diagnosis from patients with PDAC diagnosed from January 2020 to June 2024 at the Hospital Clínico Universitario de Valencia (268 patients). Resection specimens and tumours metastatic to the pancreas were excluded.

**FIGURE 1:**
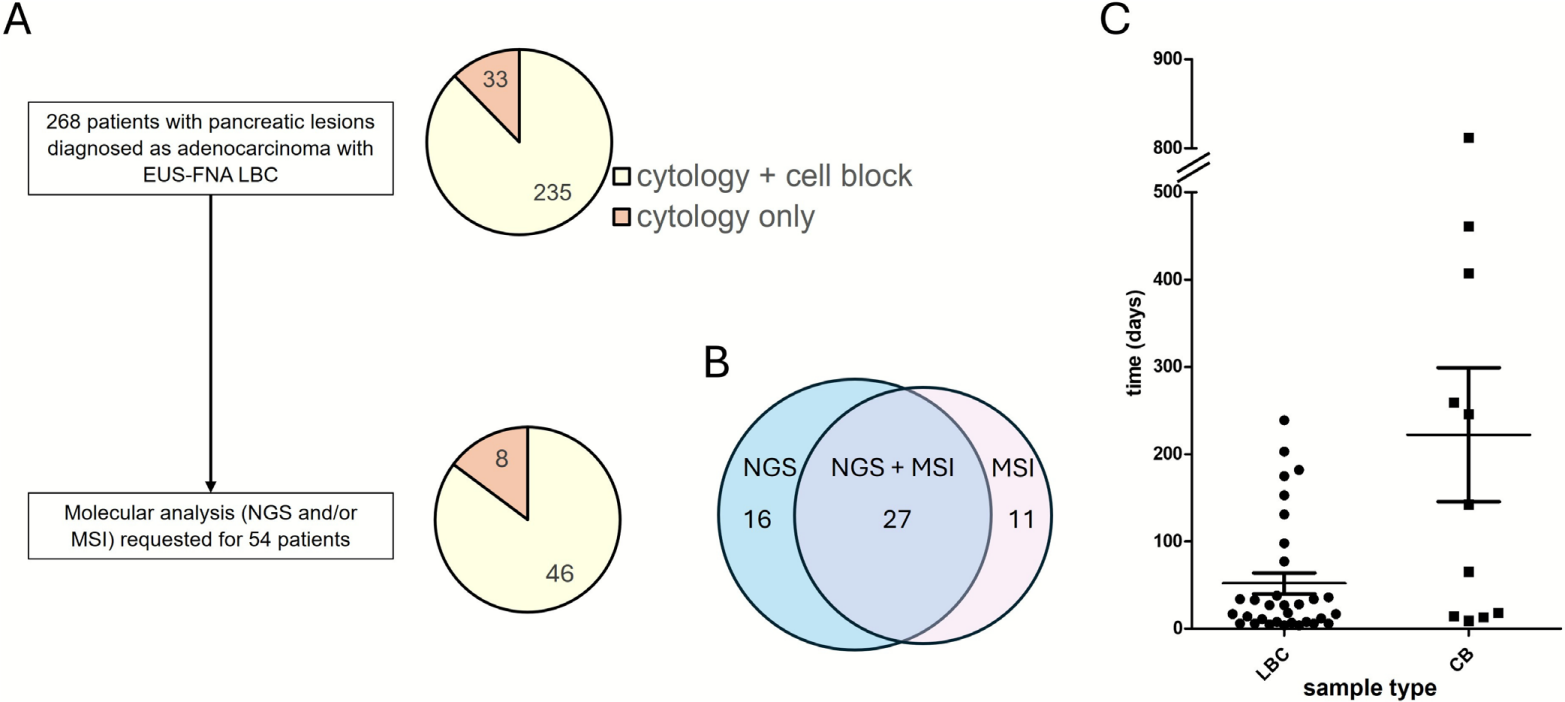
Patients and samples included in the study. A total of 268 patients were diagnosed with pancreatic adenocarcinoma using EUS‐FNA and LBC, and cell blocks were produced when possible (A). Molecular analyses were requested for 54 patients and included NGS and MSI (B). Analyses were requested at varying timepoints after pancreatic adenocarcinoma diagnosis (C). EUS‐FNA, ultrasound‐guided fine needle aspiration; LBC, liquid‐based cytology; CB, cell block.

We retrospectively analysed the quality and results of medically indicated molecular diagnoses (NGS and/or MSI) from 54 patients (Figure 1A). All patients provided informed consent for the FNA‐EUS procedure and subsequent molecular diagnosis when requested. Clinical information was obtained from medical records, and the study protocol was approved by the Institutional Review Board (2024/316).

### 2.2 EUS‐FNA and Cytology Sample Preparation

FNA of solid pancreatic mass was performed by experienced gastroenterologists during endoscopic ultrasound using a 25G needle. EUS‐FNA samples were immersed in ThinPrep CytoLyt (Hologic) solution and submitted for pathologic diagnosis.

Any visible solids were removed, transferred to cell block specific cassettes and embedded in paraffin. Cell block sections (4 μm‐thick) were stained with haematoxylin and eosin (H&E).

Cytology LBC samples were prepared following the manufacturer's instructions. Residual LBC samples were routinely stored at room temperature (RT) for 1–2 months depending on laboratory storage space requirements. Pathological diagnoses of EUS‐FNA samples from pancreatic masses were periodically reviewed in the local information system to ensure longer storage of residual ThinPrep vials with a diagnosis of malignancy.

### 2.3 Nucleic Acid Extraction and Sequencing

The decision to use residual LBC or cell block was made individually by an experienced pathologist based on sample availability and quality (percentage of neoplastic cells and presence of necrosis). Cell block samples were considered adequate with tumour cell content ≥ 10%, DNA and RNA input concentration above 1 ng/μL, more than 300 tumour cells, and a tissue area greater than 3 mm^2^. Defining strict cutoffs is more challenging for cytology specimens, but a minimum of 10% tumour cells was set. Of the 43 NGS requested, 32 were performed on LBC and 11 on cell block.

DNA and RNA extraction from cell blocks and LBC were performed using the protocols available at the time of analysis. Initially, nucleic acids were isolated using Qiagen extraction kits (cell blocks: QIAamp DNA FFPE Tissue Kit, Cat. No. 56,404 and RNeasy FFPE Kit, ID. 73,504; LBC samples: QIAamp DNA Mini Kit, ID. 51,304; RNeasy Mini Kit, ID. 74,104). For each extraction, 15‐μm sections from cell block samples (two for DNA and two for RNA) and a total of 400 μL from LBC samples (200 μL for DNA and 200 μL for RNA) were required. From May 2022 onwards, all samples (cell blocks and LBC) were processed using the Genexus FFPE DNA and RNA Purification Kit (ID A45539). For each sample, one 10‐μm cell block section or 200 μL of LBC material was used as input for both RNA and DNA extraction. Quantification of nucleic acids was carried out by fluorometry (Qubit, Thermo Fisher Scientific), either manually or automatically with the Genexus kits, and served in our clinical workflow as a pre‐sequencing quality control step.

Library preparation, template generation and sequencing were automated using Thermo Fisher Scientific proton technology. NGS analysis was conducted using various targeted commercial panels (Thermo Fisher Scientific), including DNA (somatic variants and copy number variations) and RNA (gene fusions) as they were incorporated into the Pathology department. The first panel used was the Oncomine Focus Assay (OFA), which includes 52 target genes. From 2022 onwards, the predominant panel was the Oncomine Comprehensive Assay v3 (OCA v3), including 161 genes, with occasional use of the Oncomine Precision Assay (OPA) with 50 genes and the Oncomine Childhood Cancer Research (OCCRA) panel with 203 genes. Table S1 summarises the genes covered by each panel. Quality parameters included total number of mapped reads, percentage of on‐target reads, uniformity, median absolute pairwise difference (MAPD), average read depth and average amplicon length.

Sequencing results were evaluated using Thermo Fisher Scientific software, in accordance with the sequencer used, utilising the hg19 reference genome. The databases accessed to annotate and interpret the variants found included cBioPortal, VarSome, Franklin, COSMIC, CiVIC, ClinVar, MyCancerGenome, Ensembl, OncoKB, PCT, CGI, DoCM and/or PolyPhen2. Hotspot variants with an allelic frequency ≥ 3% and non‐hotspot variants with an allelic frequency ≥ 5% were reported. The minimum read depth for a detected variant was ≥ 500X. Detected fusions were reported when the number of reads was ≥ 200. Quality cutoffs used in the analysis followed the thresholds recommended by the Thermo Fisher user manuals for each specific panel. According to the manufacturer's specifications, ≥ 95% of amplicons reach a minimum sequencing depth of 500X. The bioinformatics pipelines used were not entirely identical for all panels but shared a common foundation. All analyses were performed using Ion Torrent sequencing platforms (e.g., Ion GeneStudio S5 and Genexus) and Ion AmpliSeq technology for library preparation. Variant calling and annotation were conducted using Ion Reporter software. Each panel employs specific variant‐calling parameters optimised for its targeted gene set, variant types and clinical applications.

### 2.4 RT‐PCR Detection of Microsatellite Instability

Analysis of MSI was performed with RT‐PCR Idylla MSI assay, which detects seven markers (*ACVR2A*, *BTBD7*, *DIDO1*, *MRE11*, *RYR3*, *SEC31A* and *SULF2*). It enables rapid, automated diagnosis directly from paraffin sections (in the case of cell blocks), integrating extraction and amplification and can also be applied to centrifuged LBC samples.

### 2.5 Statistical Analysis

To compare DNA and RNA concentrations obtained from LBC and cell block samples, the two‐tailed Mann–Whitney *U* test was applied for non‐parametric data, while time dependent variables were evaluated using linear regression analysis. Given the small sample size, Fisher's exact test was employed to assess the validity of molecular analysis results between LBC and cell block samples. Data analysis was conducted using GraphPad Prism, with statistical significance set at *p* < 0.05.

## 3 Results

### 3.1 Residual LBC Samples Were the Only Available Option for Molecular Diagnosis in Some Patients

A total of 268 patients with suspicious solid pancreatic lesions subjected to EUS‐FNA cytology were diagnosed with adenocarcinoma in our Pathology department during the study period (4.5 years). The mean age at diagnosis was 69 years, and 135 patients (50.37%) were women. In all cases, LBC samples were processed for cytological diagnosis using the ThinPrep protocol. When possible (235 patients), solid material was fixed in formalin and embedded in paraffin to produce a cell block, while in 33 patients (14.04%) only cytology samples were available (Figure 1A).

At our center, NGS and MSI analysis are not currently performed routinely but rather at the oncologist's request, to determine PDAC patient eligibility for personalised therapy targeting a specific molecular alteration or for clinical trial inclusion. Current clinical practice guidelines do not recommend molecular testing in localised stages, as results do not impact therapeutic management or prognosis. Moreover, in the absence of a clinical trial, the choice of first‐line systemic therapy for patients with locally advanced or metastatic disease is not currently guided by specific biomarkers. Nevertheless, given the poor prognosis and limited therapeutic options available for PDAC, molecular testing is essential in selected patients, particularly when identifying actionable alterations may enable access to targeted therapies. In this setting, molecular analysis was requested in 54 patients (20.15%). Only residual LBC samples were available in eight of these patients (14.8%, 8/54). NGS was requested in 43 patients, microsatellite instability analysis (MSI) in 38, and both analyses in 27 patients (Figure 1B). Residual LBC or cell block was selected by an experienced pathologist depending on sample availability and quality (e.g., percentage and number of tumour cells or necrosis), and studies were not performed in parallel.

Molecular analysis was requested at varying timepoints ranging from 4 to 812 days (mean, 91.56 days) after pancreatic adenocarcinoma diagnosis and performed on either LBC or cell block samples (Figure 1C). Notably, 31.7% of molecular analyses were requested more than 2 months after adenocarcinoma diagnosis, compared with 56.1% requested during the first month afterwards.

### 3.2 Long Term Stored Residual LBC Is Adequate for DNA Extraction and Permits Molecular Diagnosis

**FIGURE 2:**
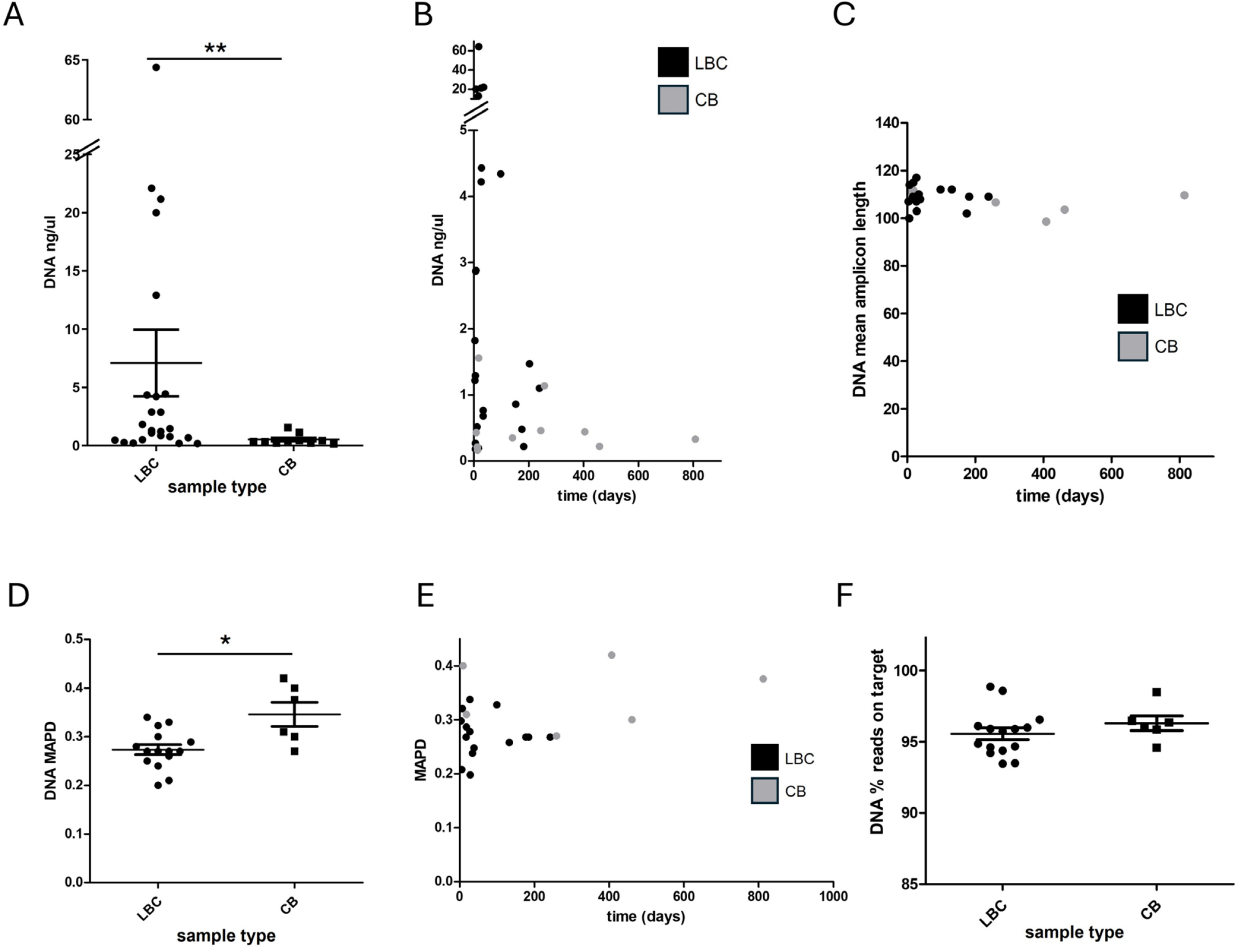
Quality and quantity of DNA derived from LBC and CB samples. DNA concentration obtained for NGS was higher from LBC samples than from cell block samples (A). DNA concentration did not vary significantly with storage time (B). Mean amplicon length did not vary with time (C). MAPD was lower in LBC than in cell blocks (D) and did not vary significantly with longer storage time (E). The percentage of reads on target was not significantly different between LBC and cell block (F). LBC, liquid‐based cytology; CB, cell block. (A, D and F) Mann–Whitney *U* test. (B, C and E) Linear regression. Error bars represent mean and standard error of the mean. **p < 0.01; *p < 0.05.

We next examined the quality and validity data available for NGS and RT‐PCR studies performed either in residual LBC or in cell block. Interestingly, DNA concentration obtained for NGS was significantly higher from LBC samples than from cell block samples (*p* = 0.0065) (Figure 2A). Although the highest concentrations were obtained with shorter storage periods, both LBC and cell block samples yielded sufficient DNA for analyses, and DNA concentration did not vary significantly with storage time (up to 239 days; 8 months) (Figure 2B). Notably, mean amplicon length indicated accurate sequencing results (mean, 108.48), with no variation over time (Figure 2C). MAPD, a metric used to assess the quality of copy number analysis, was lower in LBC than in cell blocks, indicating less noise and higher data quality (*p* = 0.0187), and did not vary significantly with longer storage time (Figure 2D,E). Finally, the percentage of reads on target was high (mean, 95.77%) and not significantly different between LBC and cell block (Figure 2F).

**FIGURE 3:**
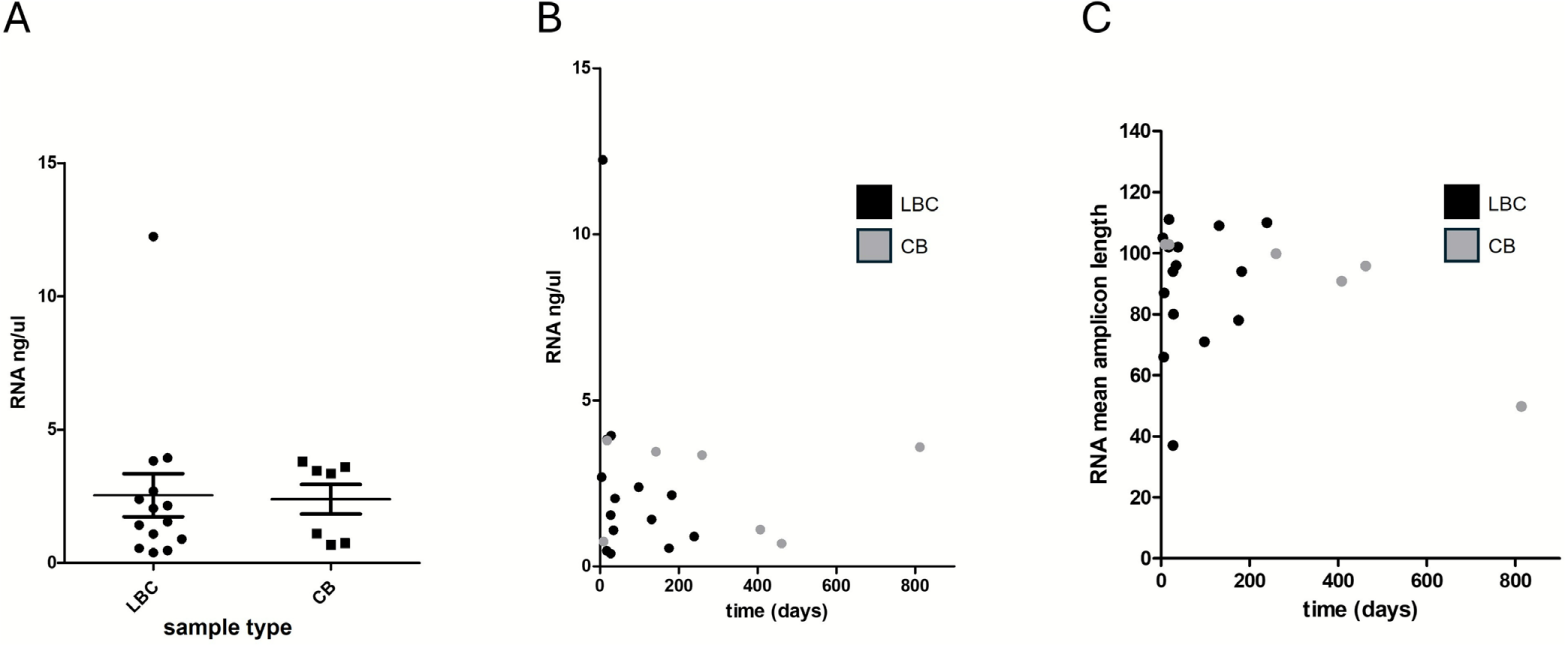
Quality and quantity of RNA derived from LBC and CB samples. RNA concentration obtained from LBC and CB samples was not significantly different (A). RNA concentration did not vary significantly with storage time (B). Mean amplicon length variation decreased significantly with storage time, but only for cell block samples (C). LBC, liquid‐based cytology; CB, cell block. (A) Mann–Whitney *U* test. (B and C) Linear regression. Error bars represent mean and standard error of the mean.

Analysis of RNA in PDAC has more limited potential, but is relevant for investigating potential fusions such as *NTRK*. RNA concentration showed no significant differences between residual LBC samples and cell blocks (Figure 3A), nor did its concentration vary significantly with storage time (Figure 3B). Mean amplicon length was lower than for DNA (mean, 89.76) in both LBC and cell blocks. Cell block samples showed a significant decrease in amplicon length with storage time (*p* = 0.02), although this trend was largely driven by a single long‐term sample (Figure 3C).

Additional quality metrics included total mapped DNA reads, DNA homogeneity, mean DNA read depth and total mapped RNA reads, with no significant differences found between LBC and cell block samples (Figure S1).

**FIGURE 4:**
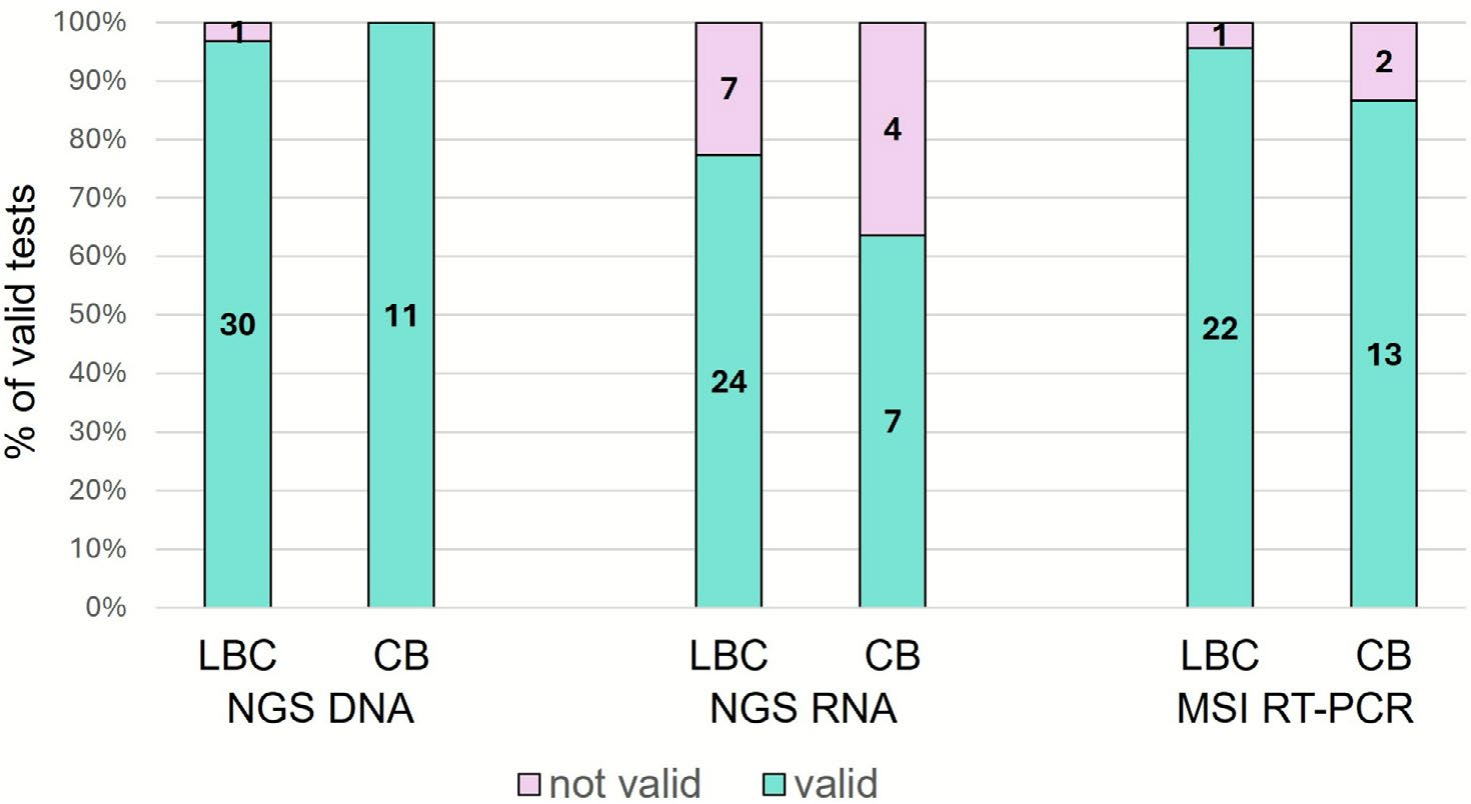
Validity of molecular analysis in LBC and cell block. There were no significant differences between sample type and validity results in NGS DNA analysis, NGS RNA analysis, or MSI RT‐PCR. LBC, Liquid‐based cytology; CB, cell block; MSI, microsatellite instability; NGS, next‐generation sequencing.

To confirm the utility of residual LBC samples for molecular analyses we next reviewed both Oncomine NGS and Idylla RT‐PCR validity results. NGS was requested in 43 patients and performed in 42 (one case not processed due to insufficient material). In NGS DNA analysis, residual LBC provided 97.62% (30/31) and cell block 100% (11/11) valid studies, with no significant differences between sample type (Figure 4A). Mutations and CNVs were identified in most patients, which further highlights the validity of the analyses. RNA was valid for NGS analysis in 77.4% (24/31) of LBC samples versus 63.6% (7/11) of cell block samples (Figure 4B). Finally, Idylla RT‐PCR to detect MSI was performed for 38 patients. DNA was invalid in only one (1/23) LBC sample and two (2/15) cell block samples (Figure 4C). No significant differences between validity and sample type were found in any kind of analysis (*p* = 1 for NGS DNA; *p* = 0.550 for Idylla MSI; *p* = 0.437 for NGS RNA).

### 3.3 Molecular Alterations Detected in Pancreatic Adenocarcinoma

**FIGURE 5:**
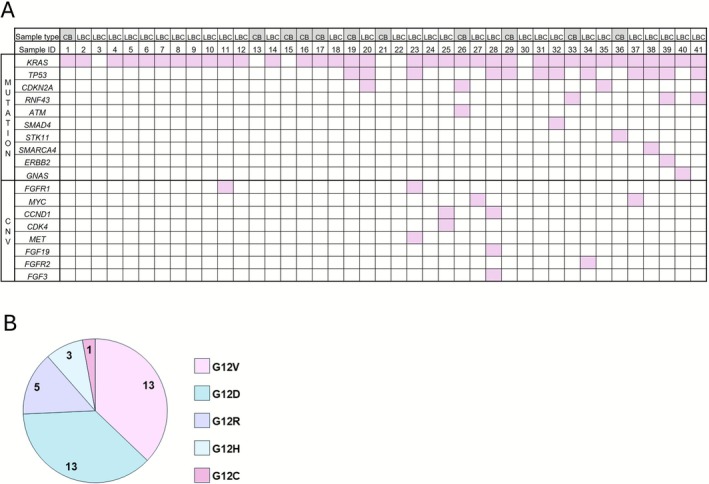
Molecular alterations detected in the study cohort. (A) NGS detected mutations and CNVs. (B) Distribution of *KRAS* mutations. LBC, liquid‐based cytology; CB, cell block.

NGS analysis was performed in 42 patients, to detect mutations, copy number variations (CNVs) and fusions. Different Oncomine panels were progressively employed, depending on their implementation in the laboratory: OFA (22 patients), OCA v3 (17), OPA (2) and OCCRA (1). Figure 5 presents all DNA alterations reported in our series of 41 patients with PDAC and valid NGS DNA. Molecular alterations were found in 35 cases (85.4%), all of which showed *KRAS* mutations. We identified additional mutations in *TP53* (29%, 12 cases), *CDKN2A* (7%, 3 cases) and *RNF43* (7%, 3 cases). Other genes were mutated in a single patient: *ATM*, *GNAS*, *SMAD4*, *STK11*, *SMARCA4* and *ERBB2* (Figure 5A). We next examined *KRAS* mutation subtypes in our series, finding G12V and G12D to be the most frequent (13 patients each). Other less common subtypes were also observed: G12R in five, G12H in three and G12C in one patient (Figure 5B). Additionally, seven patients showed CNV in the following genes: *FGFR1*, *CCND1* and *MYC* in two patients each; *MET, FGF19, FGFR2, FGF3* and *CDK4* in one patient each (Figure 5A). No fusions were detected.

In our analysis, no cases exhibited MSI. However, during the review of pancreatic LBC samples, we identified a solid pancreatic mass sampled via EUS‐FNA, where MSI was detected using RT‐PCR on residual LBC material. The patient was excluded from this study because the lesion was diagnosed as gastric adenocarcinoma metastatic to the pancreas, with MSI previously identified in the primary tumour. Nevertheless, this case underscores the potential utility of residual LBC samples for MSI detection.

## 4 Discussion

Pathological samples are often scarce in pancreatic cancer, especially in the setting of advanced, unresectable patients. These samples therefore require conscious, careful management and preservation for not only diagnosis but also for potential molecular analyses to allow access to personalised therapies for this highly aggressive tumour. Here we show that residual LBC samples from solid pancreatic lesions diagnosed as PDAC are valid for NGS and RT‐PCR. This finding is crucial given that for several patients they were the only available samples to ensure access to targeted therapies.

Our study presents a real clinical setting where molecular analysis was at the discretion of the oncologist. Remarkably, 31.7% of molecular analyses were requested more than 2 months after PDAC diagnosis, and some even up to 27 months after diagnosis. Although cell blocks are an alternative approach, they cannot be generated from all samples, as shown in our series where they were unavailable in over 14% of patients. These results underscore the importance of long‐term storage of residual LBC samples with a diagnosis of malignancy.

Current LBC storage guidelines recommend keeping samples until pathological diagnosis is completed, or longer in case of malignancy [15], but this involves systematic selection and preservation of malignant cytology samples. In our center, we periodically reviewed diagnoses of EUS‐FNA cytology, separated selected vials and stored them at RT due to space availability. Although some studies have preferentially stored samples at 4°C to reduce DNA degradation [12, 16, 17, 18], others obtained valid results storing at RT for up to 6 months [19]. Here we show that residual LBC samples remained suitable for molecular analysis at RT for up to 8 months after diagnosis. While some studies have reported DNA degradation after 9 months [20], others have shown valid results at up to 17 months storage. Given the higher instability of RNA compared with DNA, extended RT storage could compromise RNA integrity, potentially influencing the relative detectability of RNA‐based alterations. Therefore, we underscore the advantages of long‐term storage of residual LBC samples from PDAC and recommend storing them preferably at 4°C.

In our series, residual LBC samples provided valid NGS DNA analysis in 97.6% (30/31) of cases. DNA concentration was higher from residual LBC than from cell blocks and did not vary significantly with storage time. Quality parameters including mean amplicon length and percentage of reads on target were adequate. Notably, MAPD was lower in LBC than in cell blocks, indicating less noise and higher data quality, and did not vary significantly with storage time. The high percentage of mutation and CNV detection further underscores the validity of the analyses, in line with previous reports showing better results with LBC than frozen or FFPE samples [17]. DNA concentration was relatively low in most of our samples, contrasting with other studies [18], yet we report a slightly greater number of valid NGS results. This confirms that beyond DNA concentration, nucleic acid integrity is another factor affecting sequencing success, evidenced by parameters such as mean amplicon length, percentage of reads on target and MAPD.

Our series showed genomic mutations in 85.4% of cases, further confirming sequencing success and proving that samples contained an adequate tumour cell fraction. Mutations found in order of frequency were *KRAS*, *TP53* and *CDKN2A*, consistent with previous studies [17, 18, 21], with the exception of *SMAD4*, which was mutated in only one patient in our series. It is worth noting that the use of different NGS panels across time can significantly influence the observed frequency of gene alterations due to differences in gene coverage. Genes not analysed in all panels such as *SMAD4* may appear to have lower mutation frequencies, whereas those included in every panel (e.g., *KRAS*) may appear more frequently altered. In fact, the earliest cases in our series were analysed with the Oncomine OFA panel (52 genes), which did not include *SMAD4*. Since the incorporation of the OCA panel with 161 genes in 2022, we identified mutations in other potentially actionable genes, such as *ATM* or *RNF43*. The observed frequency of *KRAS* isoforms also aligns with findings from previous studies [22]. One patient showed KRAS‐G12C mutation, which occurs in 1% of pancreatic cancer cases [23] and has shown response to sotorasib [24].

Potential therapeutic targets detected by NGS RNA fusion analysis are rare in PDAC. No fusions were detected in our series, reflecting the reported low frequency of actionable gene fusions in PDAC (approximately 0.8%–1%) [25]. Nonetheless, testing for fusions such as *NTRK* is recommended in *KRAS*‐wt metastatic tumours [8]. We observed fewer valid studies for RNA (24/31), but no differences to cell blocks, further supporting long‐term storage of LBC samples. MSI is also rare in PDAC but can provide alternative treatment strategies. Most cases (22/23) were valid for RT‐PCR despite no MSI detection. However, we were able to confirm the validity of the technique in residual LBC from a pancreatic metastasis of a gastric adenocarcinoma, with confirmed MSI in the primary tumour.

As a retrospective study with a real clinical setting, the decision to use residual LBC or cell block was made by an experienced pathologist based on sample availability and quality. This is a limitation of the study, as LBC and cell block were not analysed in parallel. Our results nonetheless support the potential validity of residual LBC samples for NGS and RT‐PCR analysis and provide a rationale for future prospective studies to validate these findings.

Recent advances in molecularly targeted therapies are reshaping the therapeutic landscape of PDAC. With the recent development of KRAS inhibitors, precision medicine in PDAC is becoming increasingly feasible. In selected patients with less common molecular alterations, this could expand access to targeted therapies with tumour‐agnostic indications or proven efficacy in other malignancies, such as pembrolizumab for MSI‐high tumours. In conclusion, given that residual LBC is a valid sample for NGS and RT‐PCR and is the only available sample in some PDAC patients, preservation is crucial to guarantee patient access to personalised therapies in cases where molecular analyses are requested long after pathological diagnosis.

## Author Contributions

Raquel Antón Peñalver: data acquisition, data analysis, writing – original draft. María Ortega, José González: data acquisition, data analysis, writing – review and editing. Constanza Galindo, Clara Arnau Sancho: data acquisition. Marisol Huerta, Desamparados Roda, Valentina Gambardella, Rosana Villagrasa, Vicente Sanchiz, Andrés Peña, Elena Muñoz‐Forner, Isabel Mora Oliver, Luis Sabater, Andrés Cervantes, Antonio Ferrández: resources, writing – review and editing. Clara Alfaro‐Cervelló: conceptualization, data analysis, writing – original draft, writing – review and editing.

## Funding

The authors have nothing to report.

## Conflicts of Interest

The authors declare no conflicts of interest.

## Supporting information


**Figure S1:** Additional quality metrics of DNA and RNA derived from LBC and CB samples. Total mapped DNA reads (A), DNA homogeneity (B), mean DNA read depth (C) and total mapped RNA reads (D) were not significantly different between LBC and cell block samples. LBC, liquid‐based cytology; CB, cell block. Mann–Whitney *U* test. Error bars represent mean and standard error of the mean.

**Table S1:** Molecular NGS panels used in this study.

## Data Availability

The data that support the findings of this study are available from the corresponding author upon reasonable request.
