# Supplementary figures and images for: Finding More in Less: Precision Medicine for Pancreatic Cancer Using Residual Cytology Samples
Source: Cytopathology. 2026 Jan 26;37(3):275–83. doi: 10.1111/cyt.70055 (PMC13059547; doi:10.1111/cyt.70055)

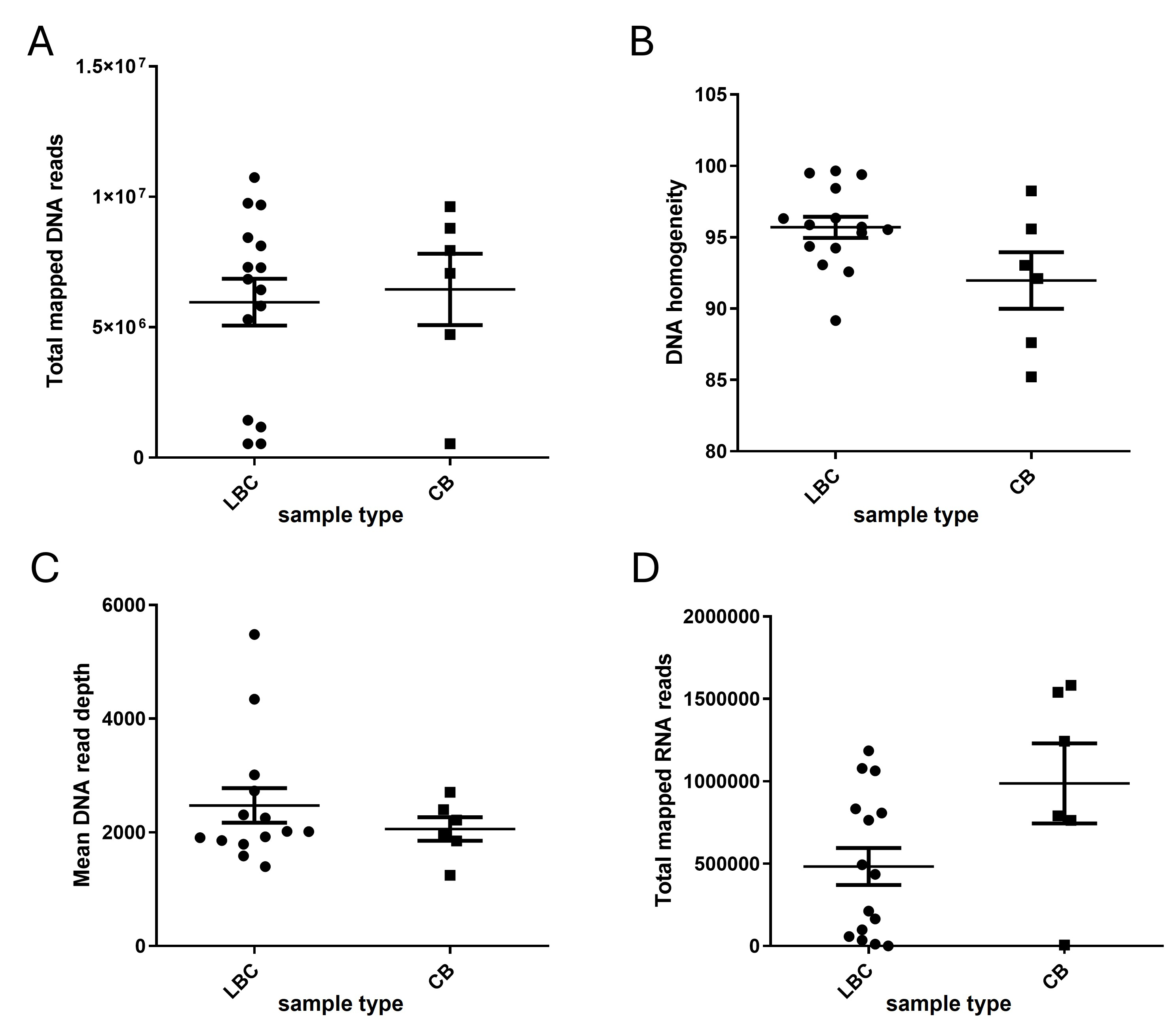

Supplement: Supplementary file 1 — Figure S1: Additional quality metrics of DNA and RNA derived from LBC and CB samples. Total mapped DNA reads (A), DNA homogeneity (B), mean DNA read depth (C) and total mapped RNA reads (D) were not significantly different between LBC and cell block samples. LBC, liquid‐based cytology; CB, cell block. Mann–Whitney U test. Error bars represent mean and standard error of the mean. [file CYT-37-275-s002.tiff]
