# Supplementary material for: Finding More in Less: Precision Medicine for Pancreatic Cancer Using Residual Cytology Samples
Source: Cytopathology. 2026 Jan 26;37(3):275–83. doi: 10.1111/cyt.70055 (PMC13059547; doi:10.1111/cyt.70055)
Supplement: Supplementary file 2 — Table S1: Molecular NGS panels used in this study. [file CYT-37-275-s001.docx]

**Supplementary Table 1. Molecular NGS panels used in the study.**

| **Oncomine Focus Assay (OFA)** |
| --- |
| **Somatic variants of hotspot regions and flanking intronic regions (± 10bp) in 35 genes** |
| AKT1, ALK, AR, BRAF, CDK4, CTNNB1, DDR2, EGFR, ERBB2, ERBB3, ERBB4, ESR1, FGFR2, FGFR3, GNA11, GNAQ, HRAS, IDH1, IDH2, JAK1, JAK2, JAK3, KIT, KRAS, MAP2K1, MAP2K2, MET, MTOR, NRAS, PDGFRA, PIK3CA, RAF1, RET, ROS1, SMO. |
| **Copy number variations (CNVs) in 19 genes** |
| AKT1, ALK, AR, BRAF, CCND1, CDK4, CDK6, EGFR, ERBB2, FGFR1, FGFR2, FGFR3, FGFR4, KIT, KRAS, MET, MYCN, PDGFRA, PIK3CA. |
| **Fusions in 23 genes** |
| ABL1, AKT3, ALK, AXL, BRAF, EGFR, ERBB2, ERG, ETV1, ETV4, ETV5, FGFR1, FGFR2, FGFR3, MET, NTRK1, NTRK2, NTRK3, PDGFRA, PPARG, RAF1, RET, ROS1. |
| **Oncomine Precision Assay (OPA)** |
| **Somatic variants of hotspot regions and flanking intronic regions (± 10bp) in 45 genes** |
| AKT1, AKT2, AKT3, ALK, AR, ARAF, BRAF, CDK4, CDKN2A, CHEK2, CTNNB1, EGFR, ERBB2, ERBB3, ERBB4, ESR1, FGFR1, FGFR2, FGFR3, FGFR4, FLT3, GNA11, GNAQ, GNAS, HRAS, IDH1, IDH2, KIT, KRAS, MAP2K1, MAP2K2, MET, MTOR, NRAS, NTRK1, NTRK2, NTRK3, PDGFRA, PIK3CA, PTEN, RAF1, RET, ROS1, SMO, TP53. |
| **Copy number variations (CNVs) in 14 genes** |
| ALK, AR, CD274, CDKN2A, EGFR, ERBB2, ERBB3, FGFR1, FGFR2, FGFR3, KRAS, MET, PIK3CA, PTEN. |
| **Fusions in 18 genes** |
| ALK, AR, BRAF, EGFR, ESR1, FGFR1, FGFR2, FGFR3, MET, NRG1, NTRK1, NTRK2, NTRK3, NUTM1, RET, ROS1, RSPO2, RSPO3. |
| **Oncomine Comprehensive Assay v3 (OCA)** |
| **Somatic variants of hotspot regions and flanking intronic regions (± 10bp) in 87 genes** |
| AKT1, AKT2, AKT3, ALK, AR, ARAF, AXL, BRAF, BTK, CBL, CCND1, CDK4, CDK6, CHEK2, CSF1R, CTNNB1, DDR2, EGFR, ERBB2, ERBB3, ERBB4, ERCC2, ESR1, EZH2, FGFR1, FGFR2, FGFR3, FGFR4, FLT3, FOXL2, GATA2, GNA11, GNAQ, GNAS, H3F3A, HIST1H3B, HNF1A, HRAS, IDH1, IDH2, JAK1, JAK2, JAK3, KDR, KIT, KNSTRN, KRAS, MAGOH, MAP2K1, MAP2K2, MAP2K4, MAPK1, MAX, MDM4, MED12, MET, MTOR, MYC, MYCN, MYD88, NFE2L2, NRAS, NTRK1, NTRK2, NTRK3, PDGFRA, PDGFRB, PIK3CA, PIK3CB, PPP2R1A, PTPN11, RAC1, RAF1, RET, RHEB, RHOA, ROS1, SF3B1, SMAD4, SMO, SPOP, SRC, STAT3, TERT, TOP1, U2AF1, XPO1. |
| **Copy number variations (CNVs) in 43 genes** |
| AKT1, AKT2, AKT3, ALK, AR, AXL, BRAF, CCND1, CCND2, CCND3, CCNE1, CDK2, CDK4, CDK6, EGFR, ERBB2, ESR1, FGF19, FGF3, FGFR1, FGFR2, FGFR3, FGFR4, FLT3, IGF1R, KIT, KRAS, MDM2, MDM4, MET, MYC, MYCL, MYCN, NTRK1, NTRK2, NTRK3, PDGFRA, PDGFRB, PIK3CA, PIK3CB, PPARG, RICTOR, TERT. |
| **Somatic variants of whole exons in 48 genes** |
| ARID1A, ATM, ATR, ATRX, BAP1, BRCA1, BRCA2, CDK12, CDKN1B, CDKN2A, CDKN2B, CHEK1, CREBBP, FANCA, FANCD2, FANCI, FBXW7, MLH1, MRE11A, MSH2, MSH6, NBN, NF1, NF2, NOTCH1, NOTCH2, NOTCH3, PALB2, PIK3R1, PMS2, POLE, PTCH1, PTEN, RAD50, RAD51, RAD51B, RAD51C, RAD51D, RB1, RNF43, SETD2, SLX4, SMARCA4, SMARCB1, STK11, TP53, TSC1, TSC2. |
| **Fusions in 51 genes** |
| AKT2, ALK, AR, AXL, BRAF, BRCA1, BRCA2, CDKN2A, EGFR, ERBB2, ERBB4, ERG, ESR1, ETV1, ETV4, ETV5, FGFR1, FGFR2, FGFR3, FGFR, FLT3, JAK2, KRAS, MDM4, MET, MYB, MYBL1, NF1, NOTCH1, NOTCH4, NTRG1, NTRK1, NTRK2, NTRK3, NUTM1, PDGFRA, PDGFRB, PIK3CA, PPARG, PRKACA. PRKACB, PTEN, RAD51B, RAF1, RB1, RELA, RET, ROS1, RSPO2, RSPO3, TERT. |
| **Oncomine Childhood Cancer Research Assay (OCCRA)** |
| **Somatic variants of hotspot regions and flanking intronic regions (± 10bp) in 86 genes** |
| ABL1, ABL2, ALK, ACVR1, AKT1, ASXL1, ASXL2, BRAF, CALR, CBL, CCND1, CCND3, CCR5, CDK4, CIC, CREBBP, CRLF2, CSF1R, CSF3R, CTNNB1, DAXX, DNMT3A, EGFR, EP300, ERBB2, ERBB3, ERBB4, ESR1, EZH2, FASLG, FBXW7, FGFR1, FGFR2, FGFR3, FLT3, GATA2, GNA11, GNAQ, H3F3A. HDAC9, HIST1H3B, HRAS, IDH1, IDH2, IL7R, JAK1, JAK2, JAK3, KDM4C, KDR, KIT, KRAS, MAP2K1, MAP2K2, MET, MPL, MSH6, MTOR, MYC, MYCN, NCOR2, NOTCH1, NPM1, NRAS, NT5C2, PAX5, PDGFRA, PDGFRB, PIK3CA, PIK3R1, PPM1D, PTPN11, RAF1, RET, RHOA, SETBP1, SETD2, SH2B3, SH2D1A, SMO, STAT3, STAT5B, TERT, TPMT, USP7, ZMYM3. |
| **Copy number variations (CNVs) in 28 genes** |
| ABL2, ALK, BRAF, CCND1, CDK4, CDK6, EGFR, ERBB2, ERBB3, FGFR1, FGFR2, FGFR3, FGFR4, GLI1, GLI2, IGF1R, JAK1, JAK2, JAK3, KIT, KRAS, MDM2, MDM4, MET, MYC, MYCN, PDGFRA, PIK3CA. |
| **Somatic variants of whole exons in 44 genes** |
| APC, ARID1A, ARID1B, ATRX, CDKN2A, CDKN2B, CEBPA, CHD7, CRLF1, DDX3X, DICER1, EBF1, EED, FAS, GATA1, GATA3, GNA13, ID3, IKZF1, KDM6A, KMT2D, MYOD1, NF1, NF2, PHF6, PRPS1, PSMB5, PTCH1, PTEN, RB1, RUNX1, SMARCA4, SMARCB1, SOCS2, SUFU, SUZ12, TCF3, TET2, TP53, TSC1, TSC2, WHSC1, WT1, XIAP. |
| **Fusions in 91 genes** |
| ABL1, ABL2, AFF3, ALK, BCL11B, BCOR, BCR, BRAF, CAMTA1, CCND1, CIC, CREBBP, CRLF2, CSF1R, DUSP22, EGFR, ETV6, EWSR1, FGFR1, FGFR2, FGFR3, FLT3, FOSB, FUS, GLI1, GLIS2, HMGA2, JAK2, KAT6A, KMT2A, KMT2B, KMT2C, KMT2D, LMO2, MAML2, MAN2B1, MECOM, MEF2D, MET, MKL1, MLLT10, MN1, MYB, MYBL1, MYH11, MYH9, NCOA2, NCOR1, NOTCH1, NOTCH2, NOTCH4, NPM1, NR4A3, NTRK1, NTRK2, NTRK3, NUP214, NUP98, NUTM1, NUTM2B, PAX3, PAX5, PAX7, PDGFB, PDGFRA, PDGFRB, PLAG1, RAF1, RANBP17, RARA, RECK, RELA, RET, ROS1, RUNX1, SS18, SSBP2, STAG2, STAT6, TAL1, TCF3, TFE3, TP63, TSLP, TSPN4A, UBTF, USP6, WHSC1, YAP1, ZMYND11, ZNF384. |
|  |
